# Marrow adipocytes inhibit the differentiation of mesenchymal stem cells into osteoblasts via suppressing BMP-signaling

**DOI:** 10.1186/s12929-017-0321-4

**Published:** 2017-02-07

**Authors:** Basem M. Abdallah

**Affiliations:** 1 Molecular Endocrinology Laboratory (KMEB), Department of Endocrinology, Odense University Hospital and University of Southern Denmark, Odense, Denmark; 2 Department of Biological Sciences, College of Science, King Faisal University, Hofuf, Saudi Arabia; 3 Faculty of Science, Helwan University, Cairo, Egypt

**Keywords:** Mesenchymal stem cells, BMSCs, Osteoblast, Adipocyte, Paracrine factors, osteoblast differentiation

## Abstract

**Background:**

Reduced bone formation is associated with increased bone marrow fat in many bone-loss related diseases including aging, post-menopause, and anorexia nervosa. Several lines of evidence suggested the regulation of osteogenesis and adipogenesis of the bone marrow-derived mesenchymal (skeletal) stem cells (BMSCs) by paracrine mediators. This study aimed to investigate the impact of adipocytes-secreted factors on the cell proliferation and osteoblast differentiation of BMSCs.

**Methods:**

Serum free conditioned medium (CM-Adipo) was collected from stromal ST2 cells-derived adipocytes. Cell viability, quantitative alkaline phosphatase (ALP) activity assay, Alizarin red staining for matrix mineralization and osteogenic gene array expression were performed to determine the effect of CM-Adipo on cell proliferation and osteoblast differentiation of primary murine BMSCs (mBMSCs). Regulation of BMPs and NF-κB signaling pathways by CM-Adipo were detected by Western blot analysis and gene reporter assay.

**Results:**

CM-Adipo showed no effect on cell viability/proliferation of primary mBMSCs as compared to CM-control. On the other hand, CM-Adipo significantly inhibited the commitment of mBMSCs into osteoblastic cell lineage in dose-dependent manner. CM-Adipo was found to dramatically inhibit the BMP2-induced osteoblast differentiation and to activate the inflammatory NF-κB signaling in mBMSCs. Interestingly, treatment of mBMSCs with the selective inhibitor of NF-κB pathway, BAY11-770682, showed to retrieve the inhibitory effect of CM-Adipo on BMP2-induced osteoblast differentiation in mBMSCs.

**Conclusions:**

Our data demonstrated that the marrow adipocytes exert paracrine inhibitory effect on the osteoblast differentiation of mBMSCs by blocking BMPs signaling in a mechanism mediated by adipokines-induced NF-κB pathway activation.

**Electronic supplementary material:**

The online version of this article (doi:10.1186/s12929-017-0321-4) contains supplementary material, which is available to authorized users.

## Background

Bone marrow-derived mesenchymal stem cells (BMSCs, also known as bone marrow skeletal stem cells) reside in the perivascular compartment of bone marrow and can differentiate into osteoblast and adipocyte cell lineages among other mesoderm cell types [1, 2]. BMSCs hold a great promise in cell-based therapy for many degenerative diseases including osteoporosis, due to their differentiation potential, immune-modulatory functions and the secretion of paracrine factors involved in endogenous tissue regenerative capacity [3, 4].

Lineage-specific differentiation of BMSCs into either osteoblasts or adipocytes is regulated by many paracrine factors including cytokines/growth factors and hormones that act to induce intercellular signaling and subsequently activate the key transcriptional factors, core-binding factor 1(CBFA1/Runx2) [5], or peroxisome proliferator-activated receptor gamma 2 (PPARγ2) [6] for osteogenesis and adipogenesis respectively.

Adipocytes-secreted adipokines and free fatty acids affect both osteoblasts and osteoclasts formation/activity and therefore mediate skeletal homeostasis [7, 8]. Marrow fat volume was observed to be increased in animal models of ovariectomy, aging and calorie restriction and in human, it was inversely correlated with BMD in the clinical conditions of aging, post-menopause, and anorexia nervosa (for review, [9–14]. Several studies attributed this increased marrow adiposity to the shifting in the differentiation capacity of BMSCs towards adipocyte versus osteoblast cell lineage, suggesting an inverse relationship between these two lineages [10, 15–17]. In vitro and in vivo studies reported a paracrine regulatory mechanism for controlling this inverse relationship between osteoblast and adipocyte differentiation of BMSCs [7, 9, 10]. For examples: adipocytes secrete factors that inhibit osteoblastogenesis and favor adipogenesis, such as sFRP-1 [18], sFRP-4, and chemerin [19] and pro-inflammatory cytokines [20].

To answer the question whether increased marrow adipocytes is one of the main contributing factor to reduce osteoblast differentiation and bone formation in osteoporosis, we aimed in this study to investigate the impact of adipocyte-secreted factors on BMSCs proliferation and osteoblast differentiation. Thus, we studied the paracrine effect of the serum free condition medium collected from stromal ST2 cell line-derived adipocytes (CM-Adipo) on the cell proliferation and differentiation of murine BMSCs. Results showed the inhibitory effect of adipocyte-secreted factors on the differentiation of mBMSCs into osteoblastic cell lineage without affecting their proliferation. Interestingly, CM-Adipo was found to block BMP2-induced osteogenesis via activating the inflammatory NF-κB pathway.

## Methods

### Cell culture

Mouse stromal cells ST2 was obtained from Leibniz Institute DSMZ-German Collection of Microorganisms and Cell Cultures (ACC 333, Braunschweig, Germany). Cells were cultured in DMEM supplemented with 10% fetal bovine serum (FBS) and 1% penicillin/streptomycin (P/S) (all purchased from Gibco Invitrogen, USA).

Mouse BMSCs were isolated from wild-type 8-weeks-old male C57BL/6 J mice as previously described [21]. In brief, the ends of mouse tibia and femur were cut and placed in special adapted Eppendorf tubes, centrifuged for 1 min at 400 g to collect the marrow cells. Cell were filtrated through a 70-μm nylon mesh filter and cultured in 175 cm^2^ flasks in RPMI-1640 medium supplemented with 12% FBS (Gibco Invitrogen, USA), 12 μM L-glutamine (Invitrogen) and 1% penicillin/streptomycin (P/S) (Gibco Invitrogen, USA). Non-adherent cells were removed after 24 h by washing with PBS, and adding 30 ml of fresh medium. Every 3 to 4 day, cells were washed, and fresh medium was added for a period of 4 weeks. After 4 weeks, cells were washed and trypsinized.

The NF-κB inhibitor, BAY11-7082, that inhibits IκBα [inhibitor of NF-κB (nuclear factor κB)α] phosphorylation in cells [22] and insulin were from Sigma-Aldrich ApS (Brondby, Denmark). Bone morphogenetic protein-2 (BMP2), recombinant PDGF-BB and recombinant murine WNT-3a were purchased from PeproTech (London, UK). The concentrations of different growth factors were selected as previously describe [23–25], or based on manual instructions.

### Collection of conditioned medium (CM)

ST2 cells and primary isolated murine BMSCs were induced to differentiate into adipocytes for 12 days as described below. To avoid any influence from the adipogenic inducer factors (i.e 1-methyl-3-isobutylxanthine (IBMX), dexamethasone and insulin) in the collected CM, medium was changed with serum free DMEM containing 1% P/S, and the adipocyte-derived CM (CM-Adipo) was collected after 24 h. Serum free control CM (CM-Control) was collected from un-differentiated ST2 cells that cultured for 12 days in basal culture medium. Collected CM was centrifuged for 10 min at 1000 rpm and aliquoted into small aliquots for different experiments. Based on the experimental setting, the serum free CM was used at either 100% or diluted with DMEM at 25% or 50%. FBS and other osteogenic inducers were added freshly to the CM upon studying the effect of CM-Adipo on osteogenesis of mBMSCs. CM-Adipo obtained from ST2 cells-derived adipocytes was used throughout this study, except otherwise stated.

### Cell proliferation study

Short-term in vitro cell growth was determined by culturing the cells at 2000 cells/well in 4 well plates in either CM-Adipo (100%) or CM-control (100%) supplemented with 2% FBS. Cells were trypsinized and counted by the hemocytometer.

### Real time-polymerase chain reaction (RT-PCR)

RNA was extracted using TRIzol according to the manufacturer’s instructions (Invitrogen) and the first strand cDNA was synthesized from 1 μg of total RNA using a Revert Aid™ H minus first strand cDNA synthesis kit (Fermentas, St Leon-Rot, Germany). RT-qPCR was performed using an ABI StepOne™ Real-TIME PCR machine (Life Technologies/Applied Biosystems) with using Fast SYBR® Green Master Mix (Applied Biosystems, California, USA). The targeted primers and reference genes are shown in Additional file 1: Table S1. The data were normalized to the geometric means of the reference genes β-actin and HPRT. The relative expression levels of each target gene were calculated using a comparative CT method [(1/(2^ΔC^_T_) formula, where ΔC_T_ is the difference between C_T_-target and C_T_-reference] with Microsoft Excel 2007®.

### PCR array analysis

Total RNA was extracted from mBMSCs induced to osteoblast differentiation in either CM-Control or CM-Adipo. Osteogenic RT^2^ Profiler™ PCR array, containing 84 osteoblast-related genes (Qiagen Nordic), was performed for each sample in triplicates using SYBR® Green quantitative PCR method on Applied Biosystems 7500 real-time PCR system and data were analyzed according to the manufacturer’s instructions.

### Adipocyte differentiation

Cells were plated at 15,000 cells/cm^2^ and cultured for 12 days in adipogenic-induction medium (AIM; DMEM supplemented with 9% horse serum, 450 μM 1-methyl-3-isobutylxanthine (IBMX), 100nM dexamethasone, 5 μg/mL insulin (Sigma-Aldrich) and 1 μM rosiglitazone (BRL 49653, Cayman Chemical, Ann Arbor, Michigan). The media was changed every three days.

### Osteoblast differentiation

Osteoblast differentiation was performed in cells plated at 10,000/cm^2^ in osteoblast-induction media (OIM) containing DMEM supplemented with 10% FBS 10 mM beta glycerophosphate (Calbiochem-Merck, Germany), 50 μg/mL L-ascorbic acid-2-phosphate (Wako Chemicals GmbH, Germany) and 10nM dexamethasone (Sigma-Aldrich, Denmark). The medium was changed every three days during induction period.

### Alkaline phosphatase (ALP) activity and quantification

ALP activity was performed after 6 days of osteoblastic induction. Cell viability was determined using the Cell Titer-Blue cell viability assay according to the manufacturer’s instructions (Promega, USA) and the viability measured at 560_Ex_/590_Em_ nm using a FLUO star Omega plate reader (BMG Laboratories). ALP activity was determined following incubation with 1 mg/ml of P-nitro phenyl phosphate in 50 mMNAHCO_3_ and 1 mM MgCl_2_ buffer (pH 9.6) at 37 °C for 20 min. The activity was stopped by addition of 3 M NaOH. The reaction absorbance was measured at 405 nm using a FLUO star Omega plate reader and ALP activity corrected for cell viability.

### Alkaline phosphatase (ALP) staining

After 6 days of osteoblast differentiation, cells were fixed with acetone/10 mM citrate buffer pH4.2 (1.5:1 ratio) at room temperature for 5 min and incubated for 1 h at room temperature with ALP substrate staining solution containing 0.2 mg/ml Naphtol-AS-TR-phosphate dissolved in distilled water (1:5) and 0.417 mg/ml Fast Red dissolved in 0.1 M Tris buffer.

### Oil Red O staining and quantification

At day 12 of adipocyte induction, cells were fixed in 4% paraformaldehyde for 10 min at room temperature and then stained with Oil Red O (0.5 g in 100% isopropanol) (Sigma, USA). Lipid accumulations were quantified by elution of Oil Red O in absolute isopropanol for 10 min at room temperature. The absorbance of the extracted dye was detected at 490 nm.

### Alizarin Red S staining and quantification

Calcium deposition, at day 12 of osteoblastic differentiation, was measured using Alizarin Red staining. Osteoblasts were fixed with 70% ice-cold ethanol for 1 h at -20 °C before addition of AR-S (40 mM; Sigma-Aldrich) dissolved in distilled water, pH 4.17. The cells were stained for 10 min at room temperature. The level of calcium deposition was quantified by elution of AR-S following incubation in 10% cetylpyridinium chloride (Sigma-Aldrich) for 1 h at room temperature. The absorbance of the eluted dye was assessed at 570 nm in a FLU Ostar Omega plate reader.

### Western blot assays

Cells were collected at specific time points post treatment, and washed in cold PBS buffer before being lysed in cell lysis buffer supplemented with protease inhibitor cocktail (Roche Diagnostics, Mannheim, Germany). Twenty μg of protein was separated on 8 to 12% NuPAGE® Novex® Bis-Tris gel systems (Invitrogen). The membrane was blocked and probed with antibodies and incubated with peroxidase-conjugated secondary antibody (Santa Cruz Biotechnology, Aarhus, Denmark). Antibodies for (total or phosphor) specific Smad1/5/8 and NF-κB p65 were obtained from Cell Signaling Technology (Leiden, Netherland).

### Luciferase reporter assay

The activation of NF-κB pathway was determined by using Cignal™ NF-κB luciferase Reporter Assay Kit (QIAGEN Ltd., Manchester, UK). HEK 293 or mBMSCs cells were cultured in 96-well plates and transfected with a mixture of NF-κB luciferase reporter negative control or positive control, along with Renilla construct (as an internal control) using Lipofectamine 2000 (Invitrogen) according to the manufacturer’s instructions. After 24 h, medium was changed with either CM-Adipo or CM-Control and cells were cultured for 24 h. Luciferase activities were determined using the Dual-Luciferase Assay System (Promega, Southampton, UK). Reporter activities were represented as arbitrary units after normalization to the internal Renilla reporter.

### Statistical analysis

All experiments were performed in 3–6 replicates and in at least 3 independent experiments. The data were presented as the mean ± SD. Students *t*-test was used for comparison between two groups. Differences were considered statistically significant at *P <0.05, and **P < 0.005.

## Results

### CM-Adipo does not affect the cell viability or cell proliferation of mBMSCs

**Figure 1:**
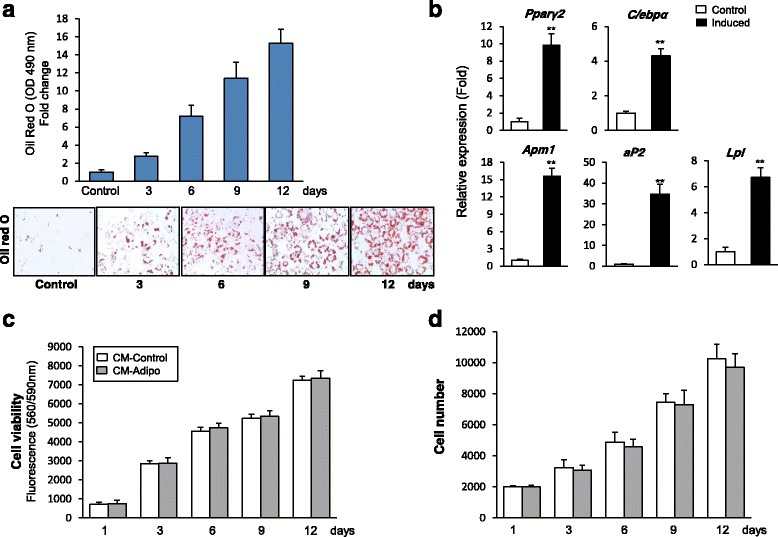
Adipocytes-derived CM has no effect on the cell viability or the cell proliferation of mBMSCs. **a** Efficient differentiation of stromal mouse ST2 cell line into adipocytes as measured by Oil red O staining and its quantification. **b** Quantitative real time RT-PCR (qPCR) analysis of the adipogenic markers mRNA expression at day 12 of the adipocyte differentiation of ST2 cells. Each target gene was normalized to reference genes and represented as fold change over non-induced control cells. **c** Effect of CM-Adipo (100%) versus CM-Control (100%) on cell viability and (**d**) cell proliferation of cultured mBMSCs. Values are mean ± SD of three independent experiments, (*p < 0.05, **p < 0.005)

We aimed to use the adipocyte-derived CM to examine the paracrine effect of adipocytes on mBMSCs proliferation and differentiation. For that purpose, the mouse stromal cell line ST2 was induced to differentiate into adipocytes for 12 days. As shown in Fig. 1a, more than 90% of ST2 cells were differentiated into adipocytes after 12 days of induction as assessed by Oil Red O staining and its quantification. In addition, the increased adipocytes formation in ST2 cells was associated with the significant upregulation of the mRNA expression of the early (*Pparγ2 and C/ebpα*) and late (*aP2, Apm1, Lpl*) adipocytic markers (Fig. 1b). We then, studied the effect of serum free collected CM-Adipo versus CM-Control on both cell viability and cell proliferation of primary mBMSCs. Neither cell viability (measured by Cell Titer-Blue) nor cell proliferation (measured by cell number) of mBMSCs was affected upon their culture for 12 days in CM-Adipo compared to CM-control (Fig. 1c&d).

### CM-Adipo inhibits the differentiation of mBMSCs into osteoblastic cell lineage

**Figure 2:**
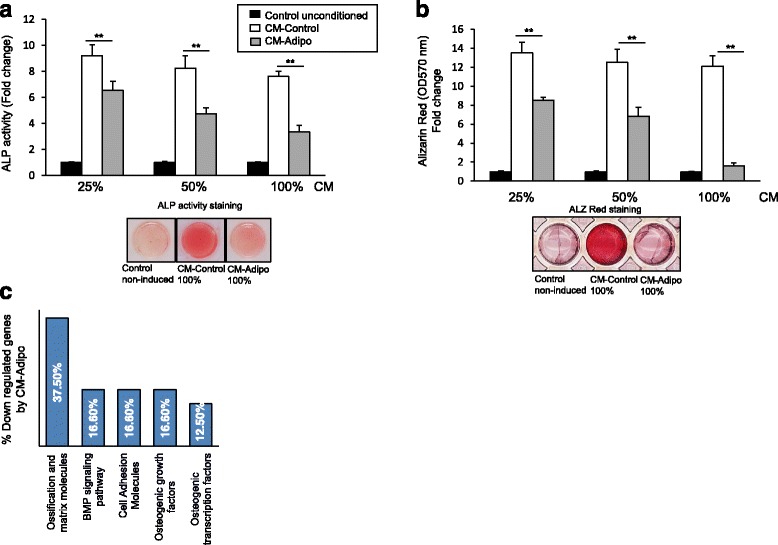
CM-Adipo exerts paracrine inhibitory effect on osteoblast differentiation of mBMSCs. **a** Dose dependent inhibitory effect of the CM-Adipo on the osteoblast differentiation of mBMSCs as measured by quantitative alkaline phosphatase activity (ALP) after 6 days of osteogenic induction and (**b**) Alizarin red staining for matrix mineralization after 12 days of induction. Representative images of the ALP activity and ALZ red staining were shown at 100% concentration of the CM. **c** Downregulated osteogenic genes in mBMSCs when cultured in CM-Adipo versus CM-Control after 6 days of osteogenic induction. Gene expression was measured by qPCR using osteogenic RT2 profiler array as described in M&M. Values are mean ± SD of three independent experiments, (*p < 0.05, **p < 0.005)

As shown in Fig. 2a&b, CM-Adipo exerted dose-dependent inhibitory effect on the osteogenesis of mBMSCs compared to CM-Control as assessed by quantitative ALP activity and matrix mineralization. Similarly, CM-Adipo obtained from primary mBMSCs-derived adipocytes showed dose-dependent inhibitory effect on ALP activity and matrix mineralization of mBMSCs during their induction into osteoblast differentiation (Additional file 2: Figure S1, A&B). Furthermore, CM-Adipo down-regulated 76.4% (≥2 fold, *p* < 0.05) of the differentially expressed osteoblastic genes during mBMSCs differentiation compared to CM-Control as measured by real time PCR-based osteogenic gene array analysis (Fig. 2c, Table 1). The down-regulated genes by CM-Adipo included, the key osteogenic transcriptional factors; *Runx2* and *Sp7* and factors involved in osteoblast differentiation and matrix mineralization, *Alp*, *Col1a1*, Ocn and integrins. We also examined the effect of CM-Adipo on the expression of the adipocytic markers of mBMSCs. As shown in Additional file 2: Figure S2, CM-Adipo did not affect the adipogenic markers of mBMSCs during their adipogenic differentiation induction as assessed by qPCR. These data support the paracrine inhibitory effect of adipocytes on osteoblast differentiate of mBMSCs, without affecting their switch between osteoblast/adipocyte differentiations.

**Table 1:** Down-regulation of osteogenic genes expression in mBMSCs treated with CM-Adipo as compared to CM-Control

Gene name	Gene symbol	Fold change
Ossification and matrix molecules		
Alkaline phosphatase, liver/bone/kidney	*Alpl*	−6.8
Bone gamma carboxyglutamate protein	*Bglap*	−3.2
Biglycan	*Bgn*	−4.5
Cadherin 11	*Cdh11*	−5.0
Chordin	*Chrd*	−4.3
Collagen type I alpha 1	*Col1a1*	−4.2
Collagen type I alpha 2	*Col1a2*	−2.4
Collagen type V alpha 1	*Col5a1*	−5.5
Secreted phosphoprotein 1 (Osteopontin)	*Spp1*	−5.8
BMP signaling pathway		
Bone morphogenetic protein 2	*Bmp2*	−6.8
Bone morphogenetic protein 7	*Bmp7*	−3.1
Bone morphogenetic protein receptor. type 1A	*Bmpr1a*	−4.5
MAD homolog 5 (Drosophila)	*Smad5*	−3.2
Cell Adhesion Molecules		
Fibronectin 1	*Fn1*	−6.2
Integrin beta 1 (fibronectin receptor beta)	*Itgb1*	−2.1
Integrin alpha 2	*Itga2*	−3.7
Integrin alpha 2b	*Itga2b*	−3.1
Osteogenic growth factors		
Fibroblast growth factor receptor 2	*Fgfr2*	−2.6
Insulin-like growth factor 1	*Igf1*	−9.2
Insulin-like growth factor I receptor	*Igf1r*	−4.1
Platelet derived growth factor. alpha	*Pdgfa*	−2.9
Osteogenic transcription factors		
Distal-less homeobox 5	*Dlx5*	−7.7
Runt related transcription factor 2	*Runx2*	−6.2
Sp7 transcription factor 7	*Sp7*	−4.3

Cells were cultured in either 100% CM-Adipo or CM-Control and induced to differentiate into osteoblasts as described in the Methods. Mouse osteogenesis RT^2^ Profiler™ PCR array with 84 osteoblast genes was performed for each cDNA sample using the SYBR® Green quantitative PCR method. Each target gene was normalized to reference genes and the differentially down-regulated genes by BMSCs in CM-Adipo were represented as fold change in the Table. Values are mean of three independent experiments

### CM-Adipo inhibits BMP2-induced osteoblast differentiation of mBMSCs

**Figure 3:**
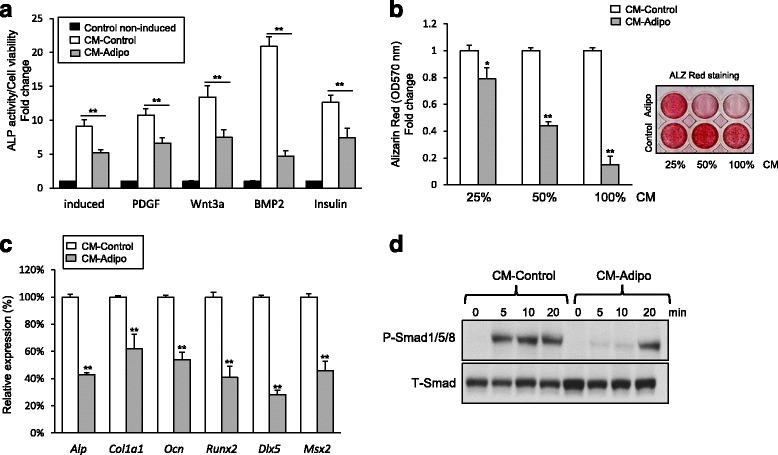
CM-Adipo inhibits BMP2-induced osteoblast differentiation of mBMSCs. **a** Studying the effect of CM-Adipo versus CM-Control on different osteogenic signaling pathways. Cultured mBMSCs were induced for osteogenesis without (control) or with regular osteogenic induction medium (induced), PDGF-BB (100 ng/ml), Wnt3a (10 ng/ml), BMP2 (100 ng/ml) and insulin (10ug/ml) in 100% of either CM-Adipo or CM-Control. ALP activity was quantified after 6 days of induction and represented as fold change over control non-induced cells. **b** Dose-dependent inhibitory effect of CM-Adipo on BMP2-induced matrix mineralization in m BMSCs. Alizarine Red staining and its quantification were performed after 12 days of induction. **c** qPCR analysis of osteoblastic gene expression in mBMSCs induced to osteoblast differentiation by BMP2 in either CM-Adipo or CM-Control for 6 days. **d** Western blot analysis of Smad1/5/8 phosphorylation in BMP2 treated mBMSCs in either CM-Adipo or CM-Control for 5-20 min. Values are mean ± SD of three independent experiments, (*p < 0.05, **p < 0.005)

To get insight into the mechanism underlying the inhibitory effect of CM-adipo on osteoblast differentiation in bone marrow, we examined the inhibitory effect of CM-Adipo on different signaling molecules known to induce osteogenesis in mBMSCs. Interestingly, CM-Adipo showed to significantly inhibit BMP2-induced ALP activity in mBMSCs by 77.5%, while the inhibitory effects on the ALP activity of other osteogenic factors including PDGF, Wnt3a and insulin were 38.5, 44.15 and 41.7% respectively (Fig. 3a). In consistent, the CM-Adipo significantly inhibited the BMP2-induced matrix mineralization in mBMSCs in dose dependent manner (Fig. 3b). Furthermore, CM-Adipo down-regulated the mRNA expression of BMP2-induced osteoblastic markers including *Runx2, Msx2, Dlx5,Ocn, Col1a1 and Alp* in mBMSCs as measured by qPCR analysis (Fig. 3c). Western blot analysis of BMP2 signaling revealed the impairment of the BMP2-induced Smad1/5/8 phosphorylation in mBMSCs upon treatment with CM-Adipo compared to CM-Control (Fig. 3d). These results demonstrated the paracrine inhibitory effect of adipocytes on BMPs signaling-induced osteogenesis in BMSCs.

### The inhibitory effect of CM-Adipo on BMP2-induced osteogenesis is mediated by NF-κB activation

**Figure 4:**
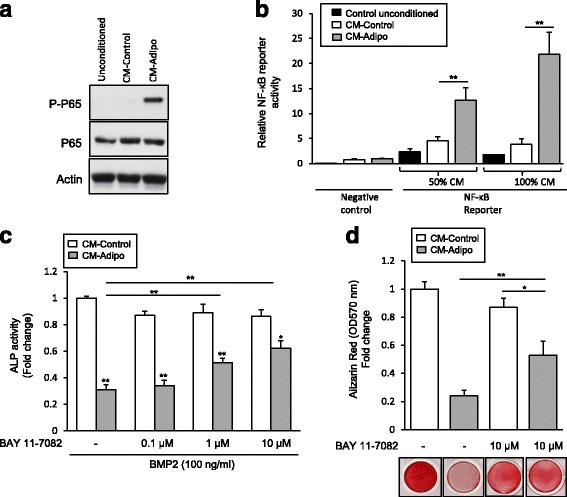
The inhibitory effect of CM-Adipo on BMP2-induced osteogenesis is mediated by activating NF-κB signaling pathway. b Western blot analysis of NF-κB subunit p-65 phosphorylation in mBMSCs cultured in CM-Adipo versus CM-Control. Cells were incubated with the CM for 30 min and cell laystes were subjected to western blot analysis. **b** CM-Adipo stimulates NF-κB signaling activity. HEK 293 cells were transfected with Cignal NF-κB Reporter negative control, or positive control. Cells were incubated with 50% and 100% CM-Control or CM-Adipo for 24 h. Dual-luciferase assays were performed, and reporter activity was represented as arbitrary units after normalization to the internal Renilla reporter. **c** Effect of NF-κb inhibitor, BAY 11-7082 on retrieving the inhibition of BMP2-induced osteoblast differentiation, as measured by ALP activity quantification and (**d**) Alizarin Red staining of matrix mineralization. Cells were treated with different concentrations of the inhibitor, 1 h prior the treatment with BMP2. ALP activity was measured after 6 days and represented as fold change after normalization to the cell viability. Alizarin Red was measured after 10 days. Values are mean ± SD of three independent experiments, (*p < 0.05, **p < 0.005)

Since NF-κB signaling was found to inhibit BMP2-induced osteoblast differentiation [26], we hypothesized that the activation of NF-κB signaling by adipokines [27] is a plausible mechanism that mediating the inhibitory effect of CM-Adipo on BMP2-induced osteogenesis. Thus, we first examined whether NF-κB signaling pathway is activated in mBMSCs by CM-Adipo. Interestingly, western blot analysis showed the stimulation of the NF-κB subunit p-65 phosphorylation in mBMSCs treated with CM-Adipo compared with CM-Control (Fig. 4a). Furthermore, CM-Adipo significantly stimulated the NF-κB reporter luciferase activity by 2.7 and 4.15 folds at 50 and 100% concentrations respectively as compared to CM-Control (Fig. 4b). Also, the same stimulatory effect of CM-Adipo on NF-κB reporter luciferase activity was obtained in transfected mBMSCs (Additional file 2: Figure S3, A). We then examined the effect of the potent NF-κB inhibitor, BAY 11-7082 (an irreversible inhibitor of IKKα) on rescuing the inhibitory effect of CM-Adipo on BMP2-induced ALP activity in BMSCs. As shown in Fig. 4c&d, BAY11-7082 significantly retrieved the inhibitory effect of CM-Adipo on BMP2-induced ALP activity and matrix mineralization in mBMSCs by 2.6 and 2.3 folds respectively. These data suggested that the inhibitory effect of CM-Adipo on BMP-induced osteogenesis is at least in part mediated via activating the NF-κB signaling.

## Discussion

In this study, we demonstrated the paracrine inhibitory effect of marrow adipocytes on the differentiation potential of mBMSCs into osteoblasts by blocking BMP2-induced osteogenesis. This effect is mediated via a mechanism involved the activation of the NF-κB signaling pathway by adipokines.

In this study, the ST2 cell line [28] was selected as a source of adipocytes for collecting the CM-Adipo due to the following reasons; a) ST2 cell line is a clone of homogenous stromal cell population that derived from the mouse bone marrow with a characteristics phenotype of pre-adipocytic cells; b) ST2 cells can efficiently differentiate into homogenous population of adipocytes; c) ST2 cells are suitable for collecting the serum free conditioned medium due to their ability to maintain their viability in serum free medium for up to 48 h.

To avoid any undesirable effects from the residual amount of adipogenic inducers (i.e Dexamethasone, IBMX and insulin) in the collected CM, we changed the adipogenic medium with serum and adipogenic inducers free medium prior to the collection of CM. This strategy eliminated the plausible competing effects between such adipogenic inducers (exist at relatively high concentrations) and the adipocytes-secreted factors (exist at low concentrations) on the osteoblast differentiation of mBMSCs. In supporting to this notion, the high concentration of dexamethasone-induced adipogenic differentiation of BMSCs was reported to suppress the proliferation of osteoblasts [29].

Our data demonstrated the paracrine inhibitory effect of CM-Adipo on osteoblast differentiation of mBMSCs. Few studies have investigated the paracrine effect of adipocytes on osteoblast differentiation. For example, Benayahu et al. [30] demonstrated the inhibitory effect of adipocyte CM on osteogenesis of marrow-derived osteoblastic cells, MBA-15 , while Maxson S, et al., [31] showed the stimulatory effect of adipocyte CM on the osteoblast differentiation of BMSCs. On the other hand, mammary adipose tissue-derived CM did not affect osteoblast differentiation of primary human osteoblasts [32]. These contradictory data can be attributed to the use of different sources of adipocytes for collecting CM. In addition, these studies apply the traditional method for collecting CM, which does not exclude the presence of adipogenic inducers. Indeed, when such inducers were omitted in a co-culture system, adipocytes were shown not only to inhibit osteoblast differentiation of BMSCs [33, 34] but also to induce their trans-differentiation into adipocytes [35].

Our data identified the BMPs signaling as the most distinct osteogenic pathway to be inhibited by the adipocytes CM. In consistence with our finding, the impairment of BMPs signaling was reported in BMSCs derived from osteoporotic postmenopausal women (the condition of increased marrow adipocytes) [36]. Recently, the activation of NF-κB was shown to suppress the BMP2-induced osteogenesis. In this mechanism, the inflammatory environment-stimulated NF-κB activity suppresses BMPs pathway via either activating the Toll-like receptor-4 and its intracellular adaptor protein My88 (TLR4/MyD88) dependent pathway [26] or interfering with DNA binding of the Smad complex [37, 38]. The interaction between BMPs and NF-κB signaling pathways was further confirmed by demonstrating that the inhibition of local inflammation is effective to promote the BMP-2 induced bone regeneration in vivo [39, 40]. Considering, that the adipocytes secret several inflammatory cytokines including IL-6, TNF-a, IL-1β, MCP1, CCL2 and PAI-1 known to be involved in the activation of the NF-κB [27, 41], our data demonstrated the stimulatory effect of CM-Adipo on the NF-κB signaling activation. In addition, we showed that the blocking of NF-κB signaling in mBMSCs rescued the inhibitory effect of CM-Adipo on BMP2-induced osteogenesis, suggesting that the inhibitory effect of CM-Adipo on osteoblast differentiation is mediated at least in part by NF-κB activation. Taken together, the inhibitory effect of CM-Adipocyte on osteogenesis is mediated by a group of secreted factors (Adipokines) rather than one individual factor and targeting the inhibition of adipocyte differentiation could be beneficiary for enhancing bone formation

## Conclusions

Marrow adiposity is inversely correlated with bone mass in many osteoporotic clinical conditions. The differentiation potential of BMSCs into either osteoblastic or adipocytic cell lineage was shown to be regulated by local and secreted factors in bone marrow microenvironment. Here, we studied the paracrine effect of adipocytes on the differentiation capacity of mBMSCs into osteoblasts. Our data demonstrated the paracrine inhibitory effect of adipocytes-secreted factors on the osteoblast differentiation of mBMSCs by blocking BMPs signaling in a mechanism mediated by the activation of NF-κB pathway. This study identified a novel mechanism that controlling the paracrine inhibitory effect of adipocytes on osteoblast differentiation of BMSCs.

## Additional files

Additional file 1: Table S1. List of primers used for qRT-PCR. (PDF 638 kb)
Additional file 2: Figure S1. CM-Adipo obtained from mBMSCs-derived adipocytes exerts paracrine inhibitory effect on osteoblast differentiation of mBMSCs. **Figure S2.** CM-Adipo does not affect the the adipogenic markers expression in mBMSCs. **Figure S3.** The stimulatory effect of CM-Adipo on NF-κb signaling pathway in mBMSCs. (PDF 937 kb)

## Abbreviations

ALP: Alkaline phosphatase
aP2: Adipocyte lipid-binding protein 2
APM1: Adiponectin
BMP: Bone morphogenetic protein
C/EBP-α: CCAAT/enhancer-binding protein alpha
Col1a1: Collagen type 1
LPL: Lipoprotein lipase
NF-κB: Nuclear factor kappa-light-chain-enhancer of activated B cells
OCN: Osteocalcin
OPN: Osteopontin
PPARγ2: Peroxisome proliferator-activated receptor gamma 2
Runx2: Runt-related transcription factor 2
sFRP: Secreted Frizzled-Related Protein 4
