# Supplementary material for: Marrow adipocytes inhibit the differentiation of mesenchymal stem cells into osteoblasts via suppressing BMP-signaling
Source: J Biomed Sci. 2017 Feb 7;24:11. doi: 10.1186/s12929-017-0321-4 (PMC5296965; doi:10.1186/s12929-017-0321-4)
Supplement: Additional file 2: Figure S1. — CM-Adipo obtained from mBMSCs-derived adipocytes exerts paracrine inhibitory effect on osteoblast differentiation of mBMSCs. Figure S2. CM-Adipo does not affect the the adipogenic markers expression in mBMSCs. Figure S3. The stimulatory effect of CM-Adipo on NF-κb signaling pathway in mBMSCs. (PDF 937 kb) [file 12929_2017_321_MOESM2_ESM.pdf]

Figure S1

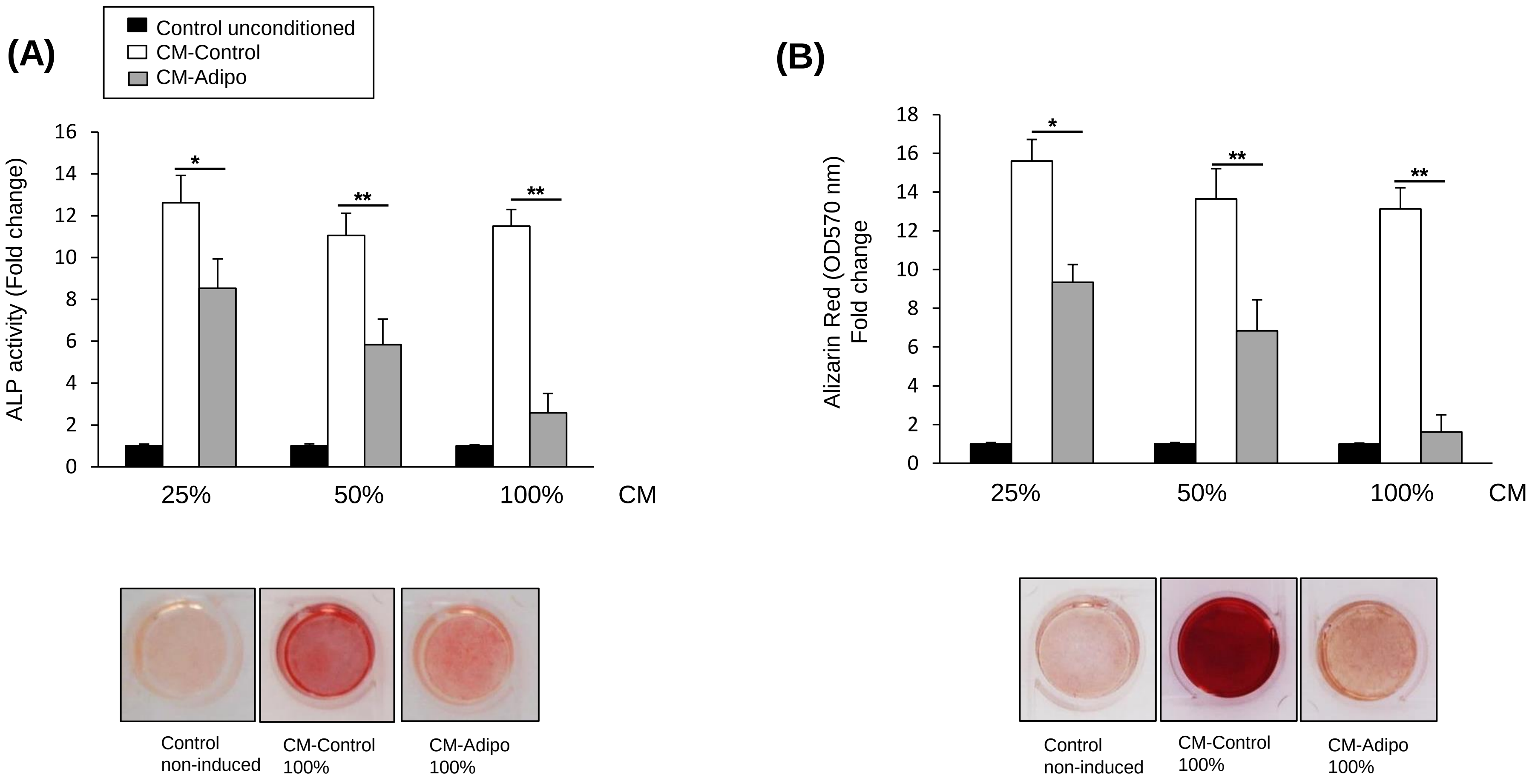

Figure S1: CM-Adipo obtained from mBMSCs-derived adipocytes exerts paracrine inhibitory effect on osteoblast differentiation of mBMSCs.

(A) Dose dependent inhibitory effect of the CM-Adipo obtained from adipocytes derived from mBMSCs on the osteoblast differentiation of mBMSCs as measured by quantitative alkaline phosphatase activity (ALP) after 6 days of osteogenic induction and (B) Alizarin red staining for matrix mineralization after 12 days of induction. Representative images of the ALP activity and ALZ red staining were shown at 100% concentration of the CM. Values are mean ± SD of three independent experiments, (*$p < 0.05$, **$p < 0.005$).

Figure S2

(A)

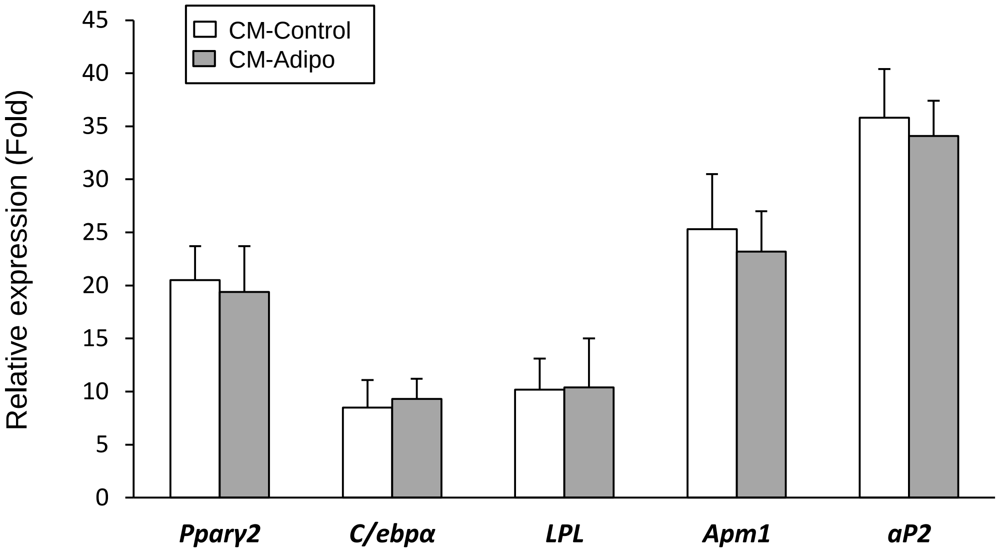

Figure S2: CM-Adipo does not affect the the adipogenic markers expression in mBMSCs

(A) Effect of CM-Adipo (100%) versus CM-Control (100%) on the adipogenic markers mRNA expression of mBMSCs at day 12 of the adipocyte differentiation as assessed by qPCR analysis. Each target gene was normalized to reference genes and represented as fold change over non-induced control cells. Values are mean ± SD of three independent experiments,

Figure S3

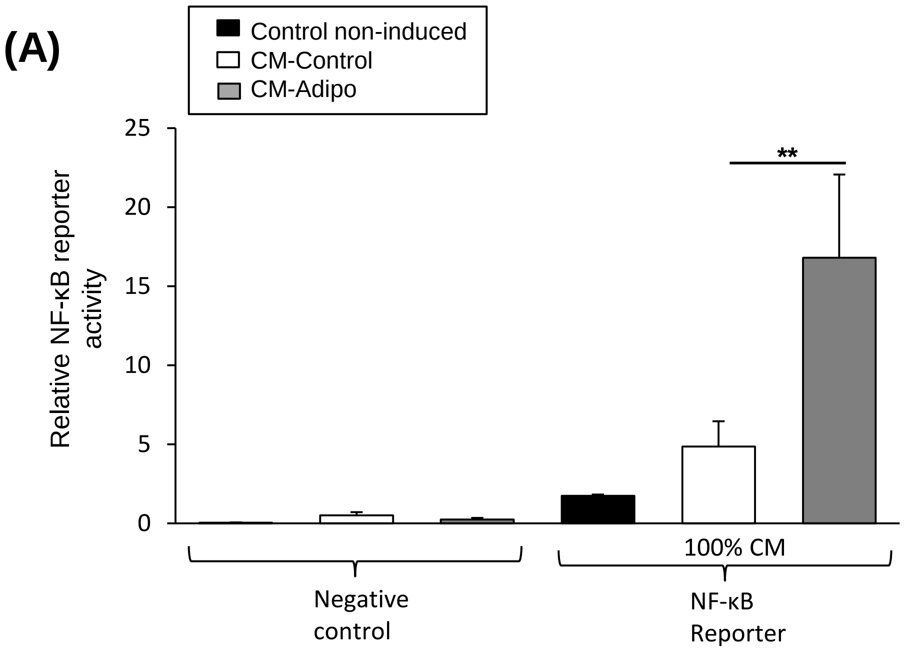

Figure S3: The stimulatory effect of CM-Adipo on NF-κb signaling pathway in mBMSCs.
(A) CM-Adipo stimulates NF-κB signaling activity in mBMSCs. Primary isolated mBMSCs were transfected with Cignal NF-κB Reporter negative control, or positive control. Cells were incubated with 100% CM-Control or CM-Adipo for 24 hours. Dual-luciferase assays were performed, and reporter activity was represented as arbitrary units after normalization to the internal Renilla reporter. Values are mean ± SD of three independent experiments, (*p< 0.05, **p< 0.005).
